# Biocontrol features of *Pseudomonas syringae* B-1 against *Botryosphaeria dothidea* in apple fruit

**DOI:** 10.3389/fmicb.2023.1131737

**Published:** 2023-03-02

**Authors:** Zihao Sun, Baihui Hao, Cuicui Wang, Shiyu Li, Yuxin Xu, Baohua Li, Caixia Wang

**Affiliations:** ^1^Shandong Engineering Research Center for Environment-Friendly Agricultural Pest Management, Shandong Province Key Laboratory of Applied Mycology, College of Plant Health and Medicine, Qingdao Agricultural University, Qingdao, Shandong, China; ^2^Shandong Provincial University Laboratory for Protected Horticulture, Shandong Facility Horticulture Bioengineering Research Center, Weifang University of Science and Technology, Weifang, Shandong, China

**Keywords:** *Pseudomonas syringae* B-1, *Botryosphaeria dothidea*, apple fruit, oxidative damage, antioxidant and defense system, salicylic acid signaling

## Abstract

Apple ring rot caused by *Botryosphaeria dothidea* is an important disease that leads to severe quality deterioration and yield loss at pre-harvest and postharvest stages. Therefore, it is urgent to develop safe and efficient measures to control this disease. The objective of the present study was to investigate the biocontrol features of *Pseudomonas syringae* B-1 against *B. dothidea* and explore its mechanism of action utilizing *in vitro* and *in vivo* assays. The results showed that *P. syringae* B-1 strongly reduced the incidence of apple ring rot and lesion diameter by 41.2 and 90.2%, respectively, in comparison to the control fruit. In addition, the control efficiency of strain B-1 against *B. dothidea* infection depended on its concentration and the interval time. *P. syringae* B-1 cells showed higher inhibitory activities than its culture filtrates on the mycelial growth and spore germination of *B. dothidea*. Moreover, *P. syringae* B-1 treatment alleviated electrolyte leakage, lipid peroxidation, and H_2_O_2_ accumulation in *B. dothidea*-infected apple fruit by increasing antioxidant enzyme activities, including peroxidase, catalase, superoxide dismutase, and ascorbate peroxidase. We also found that strain B-1 treatment enhanced four defense-related enzyme activities and stimulated the accumulation of three disease-resistant substances including phenolics, lignin, and salicylic acid (SA) in apple fruit. In addition, strain B-1 triggered the upregulated expression of defense-related genes such as PR genes (*PR1*, *PR5*, *GLU*, and *CHI*) and two genes involved in the biosynthesis of SA (*SID2* and *PAD4*) to promote the resistance potential in apple fruit. Hence, our results suggest that *P. syringae* B-1 is a promising strategy against *B. dothidea*, mainly through reducing oxidative damage, activating defense-related enzymes, accumulating disease-resistant substances, and triggering the expression of resistance-correlated genes in apple fruit.

## 1. Introduction

*Botryosphaeria dothidea*, the most destructive pathogen of apples, is responsible for preharvest and postharvest ring rot, leading to huge economic losses in apple production. In detail, the fungal pathogen infects twigs, stems, and branches in the field and could survive several years on diseased apple trees (Tang et al., 2012; Zhao et al., 2016). According to the survey by Guo et al. (2009), the incidence of apple trees with ring rot symptoms was more than 77% in the main apple-producing areas of China. Apple fruit infected by *B. dothidea* is usually 10–20% each year, though it can reach 70% under favorable conditions for fungal growth, such as wet and humid weather (Wang and Hou, 2001).

Currently, the prevention and control of apple diseases caused by fungi are implemented by eliminating the inoculum, spraying the chemical fungicides, and bagging fruit (Zhao et al., 2016; Huang et al., 2021a). As the cost of bagging is increasing yearly, the bagless cultivation of apples has become an inevitable trend in China. Nevertheless, *B. dothidea* rot on apple fruit is the main disease that needs to be overcome under the condition of bagless cultivation. Once the apple tissues were infected by *B. dothidea*, the effects of therapeutic fungicides are low, for example, the control efficiency of difenoconazole was only 55%, which is one of the most effective fungicides (Zhang et al., 2010). Moreover, these chemical fungicides can cause adverse health effects and severe environmental pollution, in addition to fungicide resistance (Ma et al., 2002; Fan et al., 2016). Thus far, many chemical fungicides have been regulated or banned for use in postharvest fruit (Casals et al., 2010). Thus, it is pressing to seek eco-friendly, effective, and safe products to control *B. dothidea* rot on apple fruit.

Numerous studies and global programs have focused on biological control products as promising alternatives to the traditional fungicides for managing plant diseases in agricultural production (Droby et al., 2016). In Europe, biological control has been advocated since 2009, and in China, a “National research program on reduction in chemical pesticides and fertilizers” was launched in 2016. Until now, many microbial antagonists have been developed and commercialized to control plant diseases (Sharma et al., 2009; Spadaro and Droby, 2016). For apple fruit, biological control products have also been applied to reduce postharvest decay, mainly by biocontrol bacteria and yeast. For example, many biocontrol bacteria, including *Bacillus* spp., *Pseudomonas* spp., and *Streptomyces* spp., were able to reduce fruit postharvest decay (Chen et al., 2016; Zhang Q. M. et al., 2016; Calvo et al., 2017). In addition, a large number of biocontrol yeasts, such as *Meyerozyma guilliermondii*, *Rhodotorula mucilaginosa*, and *Pichia membranifaciens*, were used to control fruit decay caused by fungi during postharvest storage (Li et al., 2011; Yan et al., 2018; Zhang et al., 2020; Huang et al., 2021b).

The bacterial species belonging to *Pseudomonas* genera are widely found in nature, which include species causing plant diseases, as well as biocontrol microorganisms, already registered for agricultural biocontrol (van Lenteren et al., 2017; Aiello et al., 2019). It was reported that *P. syringae* strains were effective to control fruit postharvest decays caused by various fungal pathogens, including *Penicillium digitatum* (Smilanick, 1996; Cirvilleri et al., 2005; Panebianco et al., 2015), *Monilinia fructicola*, *Rhizopus stolonifer* (Zhou et al., 1999), *Botrytis cinerea*, and *P. expansum* (Zhou et al., 2001; Errampalli and Brubacher, 2006). It is noteworthy that the strains of *P. syringae*, for example, ESC-10, ESC-11, and 742RS, were developed and commercialized by EcoScience Corporation as biocontrol agents to manage postharvest decays on multiple fruits and even tubers (Williamson et al., 2008; Al-Mughrabi et al., 2013; van Lenteren et al., 2017). However, whether *P. syringae* could effectively protect apple fruit against *B. dothidea* infection is unknown, and the possible biocontrol mechanisms need to be further explored.

The present study explored the biocontrol bacterial strain *P. syringae* B-1, which was isolated from the apple fruit collected from a commercial orchard. The strain B-1 did neither induce necrotic lesions on the fruit of apple, orange, peach, and grape nor did it have effects on the internal fruit appearance. Hence, the aims of the present study were to first investigate the antifungal potential and the efficiency of *P. syringae* B-1 against *B. dothidea in vitro* and *in vivo*. Another purpose was to explore the underlying mechanisms of strain B-1 against *B. dothidea*, such as reducing oxidative damage, activating defense-related enzymes, accumulating disease-resistant substances, and triggering the expression of resistance-correlated genes in apple fruit. These results will further enrich our understanding of the action mechanisms of *P. syringae* and provide alternatives for postharvest apple decay caused by *B. dothidea*.

## 2. Materials and methods

### 2.1. Fruit, *Pseudomonas syringae* B-1, and *Botryosphaeria dothidea*

Apple (cv. ‘Fuji’) fruit used in this study was collected from the local orchards in Shandong, China. The fruit at a commercial mature stage has not received any postharvest treatment and was kept at 4°C before being used. The apples were disinfected and washed according to the method of Sun et al. (2021). *P. syringae* B-1, isolated from the healthy apple fruit, was preserved in China Center for Culture Collection (M2015813). The bacteria were cultured in nutrient broth (NB) on a shaker at 28°C for 48 h. *B. dothidea* LXS030101, isolated from apple fruit with ring rot symptoms, was characterized by our research team based on morphological observation and molecular identification. The strain was cultured on potato dextrose agar (PDA) for routine use (Zhang Q. M. et al., 2016). The conidia of strain LXS030101 was induced with the modified method of Leng et al. (2009). The wounded young apple fruit was inoculated with mycelial plugs of *B. dothidea* and incubated under UV light (365 nm) at 25°C. After about 2 weeks, the conidia was produced and collected from the lesion around the inoculation sites.

### 2.2. Efficiency of *Pseudomonas syringae* B-1 *in vivo*

Each apple fruit was punctured with a borer to make three wounds (5 mm wide and 3 mm deep) at the equatorial region. Then, the strain B-1 suspension at different concentrations (1 × 10^5^, 1 × 10^6^, 1 × 10^7^, 1 × 10^8^, and 1 × 10^9^ CFU mL^–1^) was dripped into the wounds. Following 48 h of incubation at 25°C, the conidia suspension of *B. dothidea* (5 × 10^5^ spores mL^–1^) was added to the treated wounds with the pipette (Lu et al., 2013; Huang et al., 2021a). All fruits were placed into enclosed plastic boxes and stored at 25°C in a relative humidity of about 95%. Thirty fruits were selected as controls and were treated with sterile water and inoculated only with the pathogen suspension. For each treatment, there were three replicates with 10 apple fruits in each replicate. Disease incidence was calculated as the percentage of infected wounds, and the lesion size around the wounds was recorded at 3 and 5 days post-inoculation (dpi).

### 2.3. Effect of interval time on the efficiency *in vivo*

The effect of interval time after *P. syringae* B-1 treatment on control efficiency was determined according to the assay mentioned in Section “2.2. Efficiency of *Pseudomonas syringae* B-1 *in vivo*” A volume of 30 μL of strain B-1 suspension at 1 × 10^8^ CFU mL^–1^ was added into each wound using a pipette. After 0, 6, 12, 24, 48, and 96 h of incubation at 25°C, respectively, 30 μL of *B. dothidea* spore suspension at 5 × 10^5^ spores mL^–1^ was inoculated to the treated wound. The fruits were incubated at a relative humidity of about 95% at 25°C for 5 days. The diseased symptoms were observed; the disease incidence and lesion diameter were recorded at 5 dpi. The assay was conducted two times, and there were three replicates with 10 apple fruit in each replicate.

### 2.4. *In vitro* inhibitory effect of *Pseudomonas syringae* B-1 cells and culture filtrates

Strain B-1 cells were obtained from 2-day-old cultures in NB by centrifuging at 12,000 × *g* for 15 min and re-suspended in distilled water. The culture filtrates were prepared by a 0.22-μm polycarbonate membrane filter (Sangon Biotech, Shanghai, China). For the inhibiting mycelium growth assay, PDA plates were prepared, which contained 10% (v/v) culture filtrates or different concentrations of strain B-1 cells (1 × 10^5^, 1 × 10^6^, 1 × 10^7^, 1 × 10^8^, and 1 × 10^9^ CFU mL^–1^). The mycelial plugs (5 mm in diameter) containing *B. dothidea* grown on medium for 3 days were transferred to the PDA plates. After incubating for 5 days at 25°C, the colony diameter of *B. dothidea* was determined and the inhibition rate was calculated. For the spore germination assay, the culture filtrates or different concentrations of strain B-1 cells and the pathogen suspension were dripped into the concavity slides (Zhang Q. M. et al., 2016). Following 12 h of incubation at 25°C, the spore germination was observed using the microscope (DM2500, Leica, German). Conidia were considered germinated when the length of the germ tube was longer than the length of the conidia. For each treatment, sterile distilled water was used as the control. The assays were carried out two times with four replicates.

### 2.5. Estimation of oxidative damage

Four treatments were performed in apple fruit including control and sterile water; B-1, *P. syringae* B-1 suspension at 1 × 10^8^ CFU mL^–1^; pathogen, *B. dothidea* suspension at 5 × 10^5^ spores mL^–1^; B-1 + pathogen, strain B-1 treatment, followed by *B. dothidea* inoculation. Fruit tissues were collected in different treatments at 0, 12, 24, 48, 72, 96, and 120 h. To reveal the oxidative damage in apple fruit, electrolyte leakage, malondialdehyde (MDA), and hydrogen peroxide (H_2_O_2_) contents were analyzed. For electrolyte leakage, it was measured according to the modified method of Ji et al. (2020). A total of 0.5 g of fresh tissues were cut into small pieces and dropped into 10 mL of deionized water. After 1 h incubation at room temperature, the conductivity (C1) was determined by a conductivity meter (DDBJ-350, Inesa, China). The tissue samples were then boiled for 15 min and the conductivity (C2) was measured. The formula (C1/C2) × 100% was used to calculate the electrolyte leakage.

For MDA content, it was conducted with the assay kit (#A003) from Nanjing Jiancheng Bioengineering Institute (Nanjing, China). For the analysis of H_2_O_2_ content, 1 g fresh tissues were homogenized with 3 mL of chilled 0.2% trichloroacetic acid (≥99.0%, Sangon Biotech, Shanghai, China), and then centrifuged at 12,000 × *g* for 15 min at 4°C. According to the report of Wang et al. (2015), the supernatant was mixed with 0.1 mmol L^–1^ phosphate buffer and 1 mol L^–1^ potassium iodide (≥99.0%, Sangon Biotech, Shanghai, China), then the absorbance of the reaction system was measured at 390 nm with a spectrophotometer (Philes, T6, China). The units of MDA and H_2_O_2_ contents were expressed as mmol in 1 kg of fresh weight (mmol kg^–1^).

### 2.6. Determination of antioxidant and defense-related enzyme activities

Apple fruit was treated with *P. syringae* B-1 or sterile water according to the method described in Section “2.5. Estimation of oxidative damage” Fruit tissues were collected near the wound at different time points and were ground to powder with liquid nitrogen. For the antioxidant enzymes, activities of catalase (CAT, #A007), peroxidase (POD, #A084), superoxide dismutase (SOD, #A001), and ascorbate peroxidase (APX, #A123) were measured with the assay kits from Nanjing Jiancheng Bioengineering Institute (Nanjing, China). For the defense-related enzymes, the activities of phenylalanine ammonialyase (PAL), polyphenoloxidase (PPO), β-1,3-glucanase (GLU), and chitinase (CHI) were assayed referring to the report of Zhang Q. M. et al. (2016). The enzyme activity units are expressed on the fresh weight basis as U g^–1^.

### 2.7. Qualification of total phenolics, lignin, and hormone contents

The total phenolics content was measured with the assay kit (#A143) from Nanjing Jiancheng Bioengineering Institute (Nanjing, China). The lignin content was assayed as described by Li et al. (2019), and the absorbance of the reaction mixture was detected at 280 nm. The lignin content was expressed as A_280_ g^–1^ on a fresh weight basis. Quantification of hormones including salicylic acid (SA) and jasmonic acid (JA) in apple fruit was analyzed using the enzyme-linked immunosorbent assay (ELISA) kit from Jingmei Biotechnology (Yancheng, China). SA and JA contents were expressed as μg kg^–1^ and ng kg^–1^ on the fresh weight basis, respectively.

### 2.8. Analysis of gene expression

Fruit tissues were collected near the wounds after being treated with *P. syringae* B-1 or sterile water at 0, 12, 24, and 48 h. Total RNA was extracted from different tissue samples and the cDNA synthesis was performed using the HiScript 1st Strand cDNA Synthesis kit (Vazyme, Nanjing, China). Quantitative real-time PCR (qPCR) was conducted as reported by Huang et al. (2021a), using a Light Cycler^®^ 96 PCR Detection System (Roche, Germany). Each reaction consists of at least three biological replicates, with a 25 μL reaction volume. The transcript levels of six genes involved in the defense response and the SA pathway (*MdPR1*, *MdPR5*, *MdGLU*, *MdCHI*, *MdSIT2*, and *MdPAD4*) were analyzed with the 2^–ΔΔCT^ method (Zhang Q. M. et al., 2016; Huang et al., 2021a). Referenced gene *MdEF1a* (elongation factor 1-a) was used to normalize the expression levels of target genes. The primers for the qPCR assay are shown in Table 1.

**TABLE 1:** Specific primers used for quantitative real-time PCR (qPCR) to analyze gene expression.

Primer name	Forward primer 5’→3’	Reverse primer 5’→3’	Annealing temperature (°C)
*MdEF1*α	ACATTGCCCTGTGGAAGTT	GTCTGACCATCCTTGGAAA	53/56/58
*MdPR1*	GCAGCAGTAGGCGTTGGTCCCT	CCAGTGCTCATGGCAAGGTTTT	58
*MdPR5*	AGCAGCTTCCCTCCTCGGC	CCCAGAAGCGACCAGACC	58
*MdCHI*	TGGAGGATGGGAAAGTGC	GGGTGAGTTGGATGGGTC	58
*MdGLU*	TGCCGTAGGAAACGAAAT	TGATGGAGGAAAGGAATT	53
*MdSID2*	TTATACTTCATTCCGCTGCT	GCCTCTAATTTTCTTTGTATGCT	56
*MdPAD4*	GCTTCACCGTAAGTTACTCG	CAAGAAACTCGCAACTGTC	58

### 2.9. Statistical analysis

Data analysis was performed using the software SPSS Version 19.0. All the data consisted of at least three replicates and were represented as mean ± standard deviation (SD). Statistical differences were conducted by analysis of variance using Duncan’s multiple range test at a significance level of 0.05.

## 3. Results

### 3.1. Efficiency of *Pseudomonas syringae* B-1 against *Botryosphaeria dothidea* in apple fruit

**FIGURE 1:**
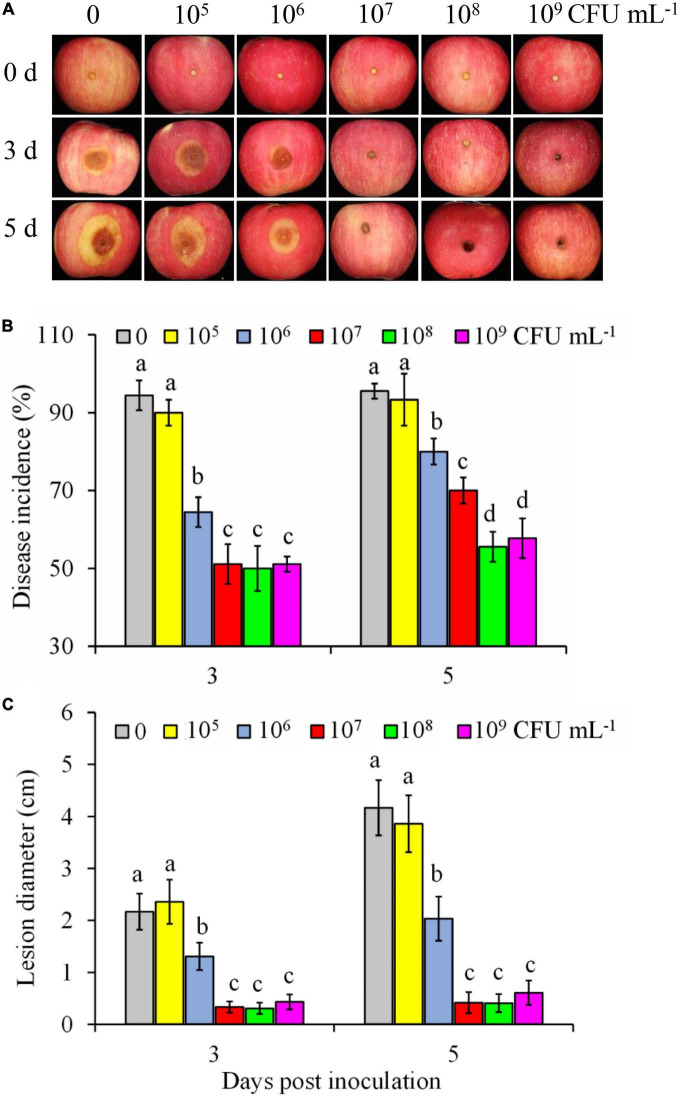
Efficiency of *P. syringae* B-1 against *B. dothidea* in apple fruit. Disease symptoms **(A)** in control and strain B-1 treated fruit at 3 and 5 days post-inoculation (dpi) with *B. dothidea*. Effect of strain B-1 on disease incidence **(B)** and lesion diameter **(C)** in inoculated apple fruit at 3 and 5 dpi. Error bars represent the SD of three replicates. Different letters above the bars indicate significant differences (*p* < 0.05) within the same panel at the same time point.

The biocontrol efficiency of *P. syringae* B-1 to control *B. dothidea* rot in apple fruit was influenced by its concentrations (Figure 1). As shown in Figure 1A, the control fruit showed rapidly developing brown lesions around the inoculation sites. In contrast, although strain B-1 at 1 × 10^5^ CFU mL^–1^ did not inhibit fruit decay caused by *B. dothidea* in fruit (Figures 1B, C), strain B-1 at 1 × 10^6^ CFU mL^–1^ to 1 × 10^9^ CFU mL^–1^ all resulted in a decrease in disease incidence (Figure 1B) and lesion diameter (Figure 1C). When the concentrations of strain B-1 were higher (>10^8^ CFU mL^–1^), their efficiency against *B. dothidea in vivo* had no significant difference. Following storage for 5 days at 25°C, strain B-1 at 1 × 10^8^ CFU mL^–1^ reduced the incidence of apple ring rot and lesion diameter by 41.2 and 90.2%, respectively, compared with the control fruit.

### 3.2. Effect of *Pseudomonas syringae* B-1 with different treatment intervals against *Botryosphaeria dothidea in vivo*

**FIGURE 2:**
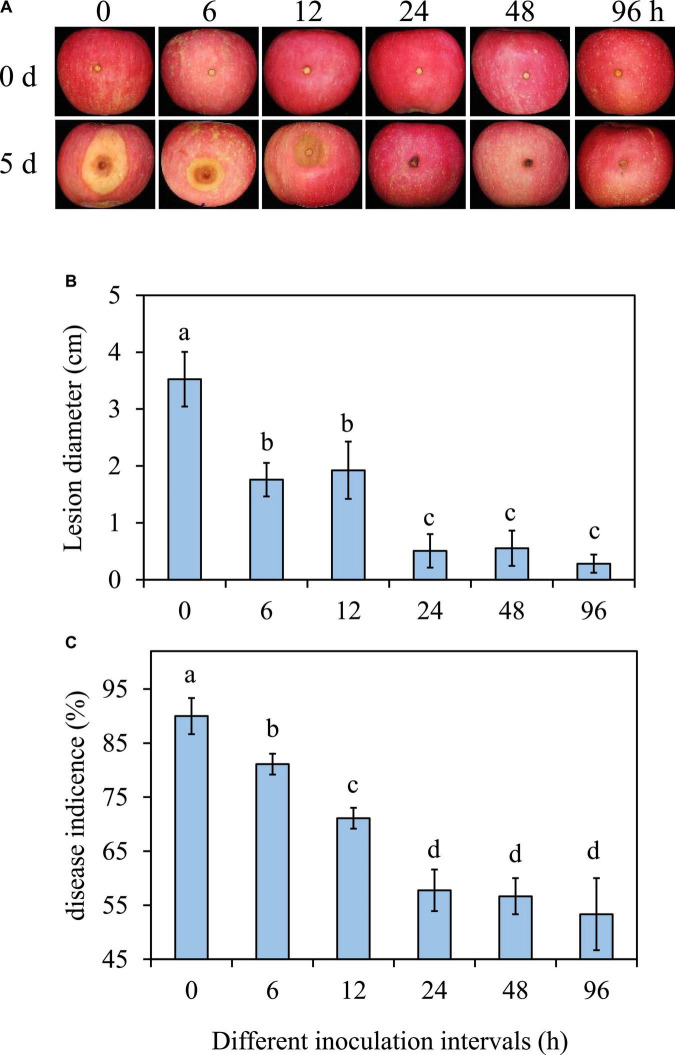
Effects of *P. syringae* B-1 with different inoculation intervals at 1 × 10^8^ CFU mL–^1^ on the disease symptoms **(A)**, lesion diameter **(B)**, and disease incidence **(C)** in inoculated apple fruit at 5 dpi. Error bars represent the SD of three replicates. Different letters above the bars indicate significant differences (*p* < 0.05) between the different inoculation intervals.

In comparison to the 0 h treatment, the diameter and incidence of *B. dothidea* rot reduced when the interval time between *P. syringae* B-1 treatment and the pathogen inoculation was 6 h or longer (Figure 2). The efficiency of strain B-1 was improved with the extension of interval time from 6 to 24 h. The expansion of decay symptoms was inhibited at the interval time of 24 h, as the incidence of apple ring rot and lesion diameter in strain B-1 treated fruit were reduced by 30.8 and 85.6%, respectively, compared with the 0 h treatment (Figure 2). There was no obvious difference in the incidence and lesion size of apple ring rot at the interval time from 24 to 96 h (Figures 2B, C).

### 3.3. *Pseudomonas syringae* B-1 inhibited *B. dothidea in vitro*

**FIGURE 3:**
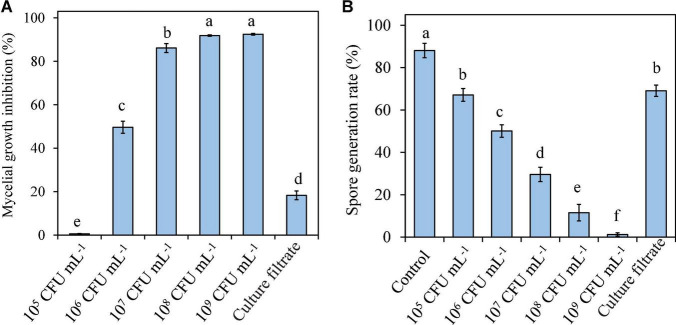
Effects of *P. syringae* B-1 cells and culture filtrate on mycelial growth inhibition **(A)** and spore generation **(B)** of *B. dothidea in vitro.* Strain B-1 cells were used at 1 × 10^5^ CFU mL–^1^ to 1 × 10^9^ CFU mL–^1^, and sterile distilled water was used as the control. Each column represents the mean and vertical bars represent the SD of four replicates. Different letters above the bars indicate significant differences (*p* < 0.05) between the control and the different treatments of strain B-1.

*Pseudomonas syringae* B-1 cells ranging from 1 × 10^6^ CFU mL^–1^ to 1 × 10^9^ CFU mL^–1^ and culture filtrates all exhibited inhibitory effects on the mycelial growth of the fungal pathogen at 25°C. When the concentration of strain B-1 cells reached 1 × 10^8^ CFU mL^–1^, the mycelial growth of *B. dothidea* was almost completely inhibited. Moreover, strain B-1 culture filtrates showed lower antifungal activity against *B. dothidea* than the bacterial cells at 1 × 10^6^ CFU mL^–1^, with 18.28 and 49.64% mycelial growth inhibition, respectively (Figure 3A).

The effect of *P. syringae* B-1 on *B. dothidea* spore germination was also determined by mixing strain B-1 cells or culture filtrates with *B. dothidea* conidia. As shown in Figure 3B, the spore germination rate decreased as the concentration of strain B-1 cells increased, which indicated that the inhibitory efficiency of strain B-1 cells against the spore germination of *B. dothidea* depended on its concentration. At 12 h of incubation, the germination rate of control reached 88.07%; however, the bacterial cells at 1 × 10^9^ CFU mL^–1^ showed the lowest germination rate with only 1.23%. Similarly, *P. syringae* B-1 culture filtrate showed less inhibition on spore germination than its cells ranging from 1 × 10^6^ CFU mL^–1^ to 1 × 10^9^ CFU mL^–1^ (Figure 3B).

### 3.4. *Pseudomonas syringae* B-1 reduced the oxidative damage in apple fruit

**FIGURE 4:**
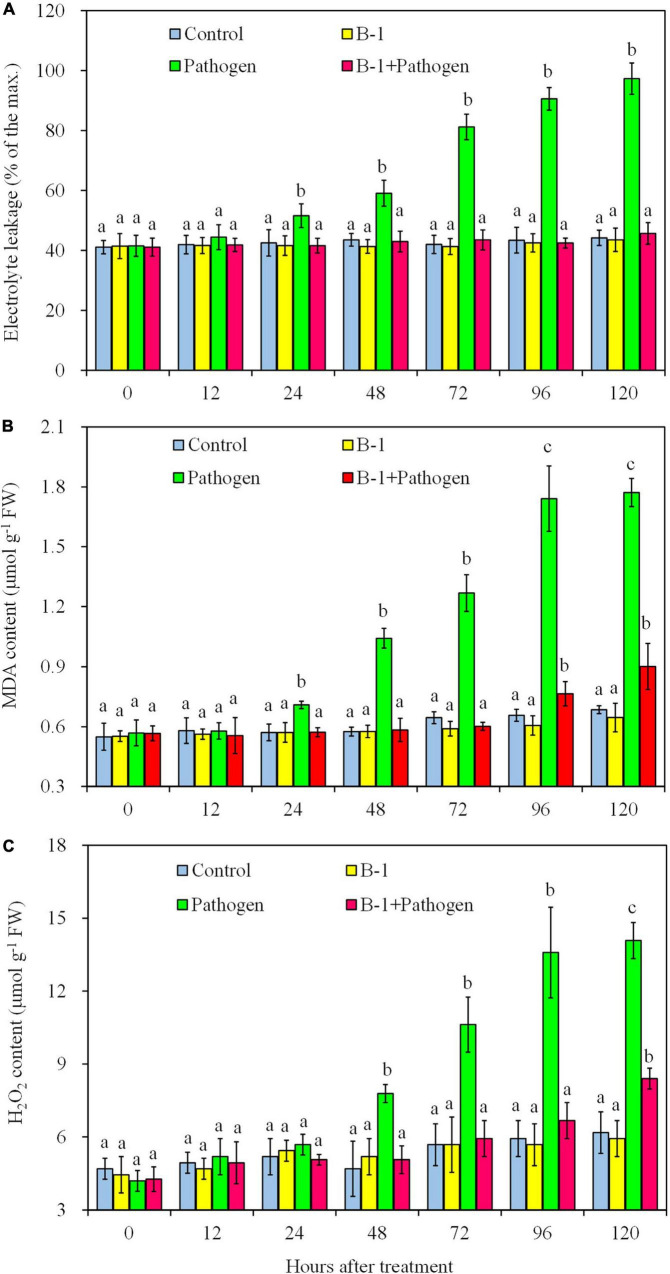
The electrolyte leakage **(A)**, MDA content **(B)**, and H_2_O_2_ content **(C)** in apple fruit with four treatments stored at 25°C. Each data represents the mean ± SD of three replicates. Different letters above the bars indicate significant differences (*p* < 0.05) at the same time point.

To analyze the effect of *P. syringae* B-1 on oxidative damage in apple fruit, the electrolyte leakage, MDA, and H_2_O_2_ contents were determined in four different treatments (Figure 4). During the storage, no statistical differences in the three factors were detected between strain B-1 treated fruit and the control. The level of electrolyte leakage rose in the fruit inoculated with *B. dothidea* from 24 h post-inoculation (hpi) and attained the maximum leakage of 97.3% at 120 hpi. However, the level of leakage in strain B-1 plus *B. dothidea*-treated fruit was only 45.7% at 120 hpi, which had no difference compared with the control fruit (Figure 4A).

In the fruit only inoculated with *B. dothidea*, MDA content increased from 24 hpi, which was up to 164.9 and 173.1% more than the control at 96 and 120 hpi, respectively (Figure 4B). In strain B-1 plus *B. dothidea*-treated fruit, although the MDA content rose from 96 hpi, it was only 16.4 and 31.6% more than the control at 96 and 120 hpi, respectively. Consistent with the level of electrolyte leakage and MDA content, the H_2_O_2_ level sharply increased in *B. dothidea*-inoculated fruit from 48 hpi and reached the highest value of 14.08 mmol kg^–1^ at 120 hpi. Nevertheless, in the fruit treated with strain B-1 plus pathogen, the level of H_2_O_2_ enhanced slowly and showed no difference compared with the control until 96 hpi, with only 0.90 mmol kg^–1^ at 120 hpi (Figure 4C).

### 3.5. *Pseudomonas syringae* B-1 affected the antioxidant enzyme activities in apple fruit

**FIGURE 5:**
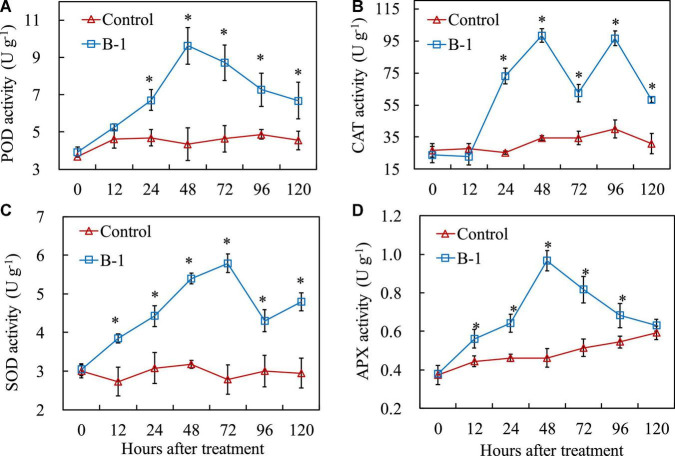
The activities of POD **(A)**, CAT **(B)**, SOD **(C)**, and APX **(D)** in apple fruit treated with *P. syringae* B-1 at 1 × 10^8^ CFU mL–^1^ stored at 25°C. Each data represents the mean ± SD of three replicates. Asterisks (*) denote a significant difference (*p* < 0.05) between the strain B-1 treatment and the control apple fruit at the same time point.

To reveal the effect of *P. syringae* B-1 on the apple fruit, the activities of the four antioxidant enzymes were analyzed (Figure 5). During storage, POD activity in apple fruit varied as time progressed (Figure 5A). An ascending tendency of POD activity was observed in fruit treated with *P. syringae* B-1 from 24 h and the peaked was at 48 h with 2.21-fold higher than the control. POD activity in strain B-1 treated fruit decreased from 72 h; however, it remained higher than the control during the whole storage. The result of Figure 5B showed that CAT activity was affected by *P. syringae* B-1 treatment. In strain B-1 treated fruit, the activity of CAT began to rapidly increase from 24 h and reached the peak at 48 h of storage, which was more than 2.85-fold higher compared with the control. Then, the enzyme activity in strain B-1 treated fruit decreased until 72 h, but CAT activity quickly rosed again and the second peak was at 96 h, which was 2.41-fold higher than the control.

In contrast to the control, *P. syringae* B-1 treatment also increased the activities of SOD and APX in apple fruit during storage (Figures 5C, D). The activity of SOD in fruit treated with strain B-1 rose steadily from 12 h and reached the peak at 72 h with 5.79 U g^–1^. Although the enzyme activity declined from 96 h, SOD activity induced by strain B-1 was still higher than the control throughout the storage period (Figure 5C). As shown in Figure 5D, APX activity was induced by stain B-1 from 12 h and peaked at 48 h, which was 2.09-fold higher than the control. Then, the enzyme activity declined rapidly and it even reduced to the control level at 120 h.

### 3.6. *Pseudomonas syringae* B-1 induced the defense-related enzyme activities in apple fruit

**FIGURE 6:**
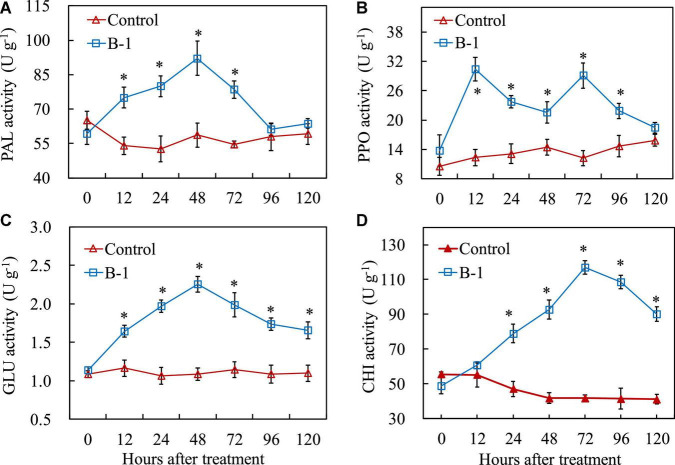
The activities of PAL **(A)**, PPO **(B)**, GLU **(C)**, and CHI **(D)** in apple fruit treated with *P. syringae* B-1 at 1 × 10^8^ CFU mL–^1^ stored at 25°C. Each data represents the mean ± SD of three replicates. Asterisks (*) denote a significant difference (*p* < 0.05) between the strain B-1 treatment and the control apple fruit at the same time point.

In addition to an enhancement of antioxidant enzyme activities by *P. syringae* B-1 treatment, Figure 6 shows that the four defense-related enzyme activities were induced by *P. syringae* B-1 in apple fruit. For PAL and PPO, the activities increased from 12 h in the fruit treated with strain B-1 and they reached the highest values, 1.57-fold and 2.47-fold higher than the control, respectively. After that, the two enzymes’ activities induced by strain B-1 decreased quickly, and they were not different from the control at 96 h for PAL and 120 h for PPO, respectively (Figures 6A, B).

Figures 6C, D show that GLU and CHI activities were affected by *P. syringae* B-1 treatment in apple fruit. The GLU activity in fruit treated with strain B-1 showed a sharp increase and peaked at 48 h. Thereafter, the enzyme activity decreased from 72 to 120 h; however, it remained higher than the control during the storage phase (Figure 6C). The change of CHI activity induced by strain B-1 was similar to GLU activity. The enzyme activity rose within 24 h after strain B-1 treatment and reached the highest level at 72 h. Then, CHI activity declined from 96 h after treatment, and it was still 2.19-fold higher than the control at 120 h (Figure 6D).

### 3.7. *Pseudomonas syringae* B-1 promoted the total phenolics, lignin, and hormone content in apple fruit

**FIGURE 7:**
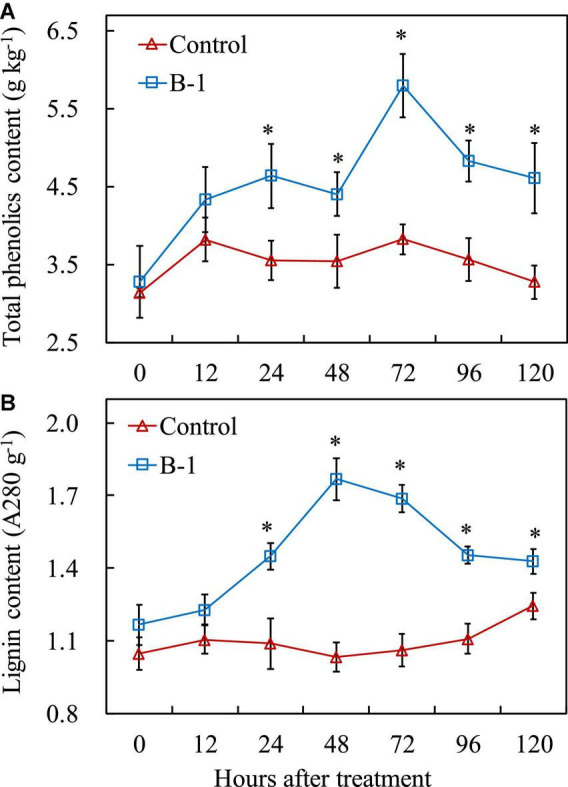
The content of total phenolics **(A)** and lignin **(B)** in apple fruit treated with *P. syringae* B-1 at 1 × 10^8^ CFU mL–^1^ stored at 25°C. Each data represents the mean ± SD of three replicates. Asterisks (*) denote a significant difference (*p* < 0.05) between the strain B-1 treatment and the control apple fruit at the same time point.

To get a better insight into the impact of *P. syringae* B-1 on apple fruit and the mechanisms of induced protection against *B. dothidea*, several metabolites and hormones involved in pathogen resistance were measured. Generally, the contents of total phenolics and lignin contents changed slowly in the control fruit, whereas they were greatly induced in strain B-1 treated fruit. As indicated in Figure 7A, strain B-1 enhanced the accumulation of total phenolics from 24 h (1.31-fold) after treatment during the storage period, and reached the maximum level at 72 h with 1.52-fold higher than the control. In addition, strain B-1 promoted the lignin content from 24 h after treatment compared with the control. Then, the lignin content in strain B-1 treated fruit peaked at 48, with 1.71-fold higher than the control. After that, it decreased only slightly and maintained a higher level throughout the storage (Figure 7B).

**FIGURE 8:**
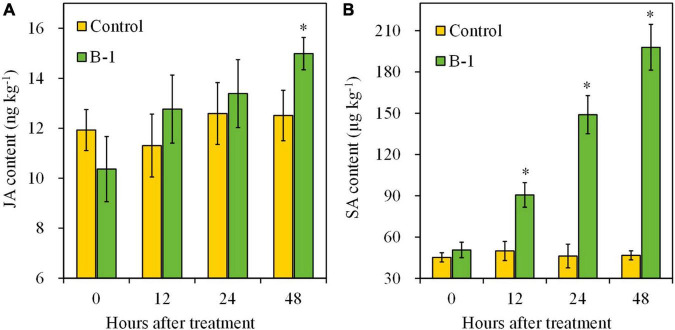
The content of JA **(A)** and SA **(B)** in apple fruit treated with *P. syringae* B-1 at 1 × 10^8^ CFU mL–^1^ stored at 25°C. Each data represents the mean ± SD of three replicates. Asterisks (*) denote a significant difference (*p* < 0.05) between the strain B-1 treatment and the control apple fruit at the same time point.

Regarding plant hormones, the contents of SA and JA were determined, with or without strain B-1 treatment. During the storage, SA and JA levels had no obvious changes in the control fruit (Figure 8). Strain B-1 treatment slightly enhanced the accumulation of JA at 48 h in apple fruit, with a 1.20-fold higher compared with the control. Nevertheless, the result of Figure 8B illustrated that strain B-1 treatment enhanced the SA levels compared with the control. The content of SA increased from 12 h and continued to accumulate, which was 4.24-fold higher than the control.

### 3.8. *Pseudomonas* s*yringae* B-1 activated the upregulated expression of PR genes and SA biosynthesis-related genes in apple fruit

Given the increased SA content in strain B-1-treated fruit, we next determined the expressions of genes involved in defense response (*MdGLU* and *MdCHI*) and SA pathway (*MdPR1*, *MdPR5*, *MdSID2*, and *MdPAD4*) by quantitative PCR to reveal the biocontrol mechanism of *P. syringae* B-1. *PR1* and *PR5* are widely used molecular markers that correlate with SA signaling activation (Zhang Q. M. et al., 2016). Meanwhile, *SID2* (SA induction deficient 2) and *PAD4* (phytoalexin deficient 4) are the genes related to SA biosynthesis (Huang et al., 2021b).

**FIGURE 9:**
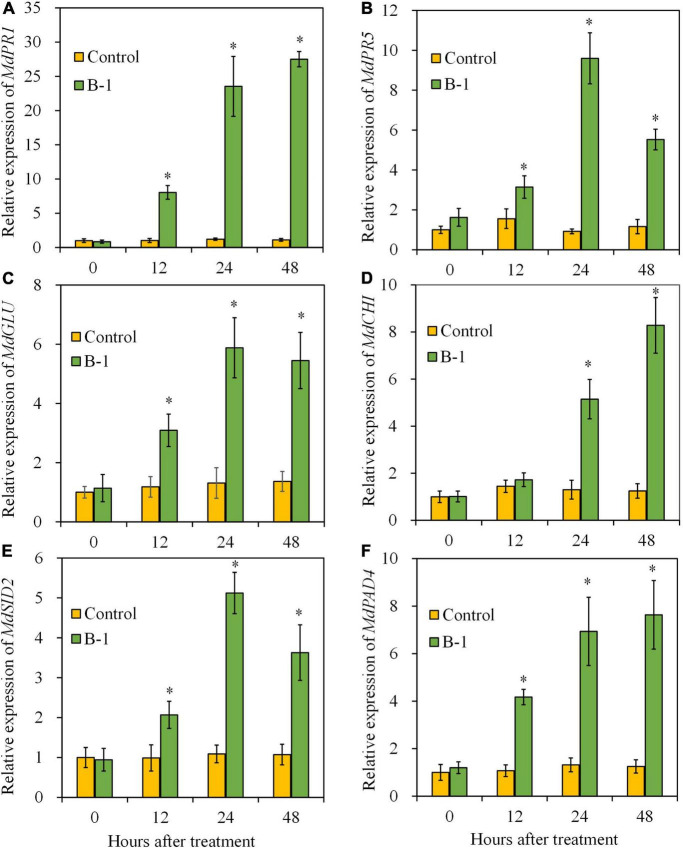
The gene expressions of *MdPR1* **(A)**, *MdPR5* **(B)**, *MdGLU* **(C)**, *MdCHI* **(D)**, *MdSID2* **(E)**, and *MdPAD4* **(F)** in apple fruit treated with *P. syringae* B-1 at 1 × 10^8^ CFU mL–^1^ stored at 25°C. Each data represents the mean ± SD of three biological replicates. Asterisks (*) denote a significant difference (*p* < 0.05) between the strain B-1 treatment and the control apple fruit at the same time point.

Compared with the control, the transcript level of *MdPR1* in strain B-1 treated fruit upregulated significantly at 12 h, and then reached the maximum at 48 h, which was 27.50-fold higher than that in the control fruit (Figure 9A). As shown in Figure 9B, the transcript level of *MdPR5* in fruit treated with strain B-1 peaked the highest at 24 h, with 9.60-fold higher than the control. Similarly, strain B-1 treatment upregulated the expression of *MdSID2* and *MdGLU* at 12–48 h, which exhibited similar trends with *MdPR5* (Figures 9C, E). The result of Figure 9F indicated that the expression of *MdPAD4* was triggered by strain B-1, and its transcript level was 7.63-fold higher than the control at 48 h. For *MdCHI*, compared with the control, the transcript level was activated by strain B-1 and showed 5.15- and 8.28-fold increases at 24 and 48 h, respectively (Figure 9D).

## 4. Discussion

Biological control is a possible and safe strategy for chemical fungicides to manage the postharvest decay in fruit and tuber (Droby et al., 2009). Previous studies indicated that many strains of the *Pseudomonas* genus, including *P. syringae*, are effective in controlling multiple postharvest decays caused by *B. cinerea*, *M. fructigena*, and *P. digitatum* rot (Zhou et al., 2001; Panebianco et al., 2015; Aiello et al., 2019). In this research, we demonstrated for the first time that *P. syringae* B-1 could effectively control postharvest apple ring rot by reducing the disease incidence and lesion size in fruit during the whole storage. The pretreatment of strain B-1 alone showed no effect on the surface color, weight loss, firmness, or total soluble solids content of apple fruit (data not shown). Given these promising results, we further investigated the possible mechanisms of *P. syringae* B-1-induced biocontrol against *B. dothidea*.

Our findings revealed that the ability of *P. syringae* B-1 to control apple ring rot depended on its concentration, and 1 × 10^6^ CFU mL^–1^ strain B-1 was its threshold concentration to control *B. dothidea* effectively. This also exists in other antagonists for their control efficiency. For example, *M. guilliermondii* at high concentration has better efficiency against blue mold decay in pear fruit (Yan et al., 2018). Moreover, we found the time interval between *P. syringae* B-1 pretreatment and *B. dothidea* infection was another important element for its efficiency *in vivo*. Previous studies also showed that the biocontrol efficiency was closely related to the interval time after antagonist treatment (Lu et al., 2013; Huang et al., 2021b), which aligned with our current finding. Furthermore, *P. syringae* B-1 showed a protective rather than curative effect to manage apple ring rot, which agreed with the biocontrol effect of *Clavispora lusitaniae* 146 and *Streptomyces* sp. H4, two agents against postharvest green mold and anthracnose, respectively (Perez et al., 2019; Li et al., 2021).

The action mechanisms of biocontrol bacteria to manage postharvest decays are complicated (Rocío et al., 2021), the understanding of which, however, is pivotal to registering and commercializing a biocontrol product (Droby et al., 2016). In this research, we analyzed the antagonist effects of *P. syringae* B-1 using live cells and culture filtrates. Overall, strain B-1 culture filtrates showed lower but notably inhibitory activity on *B. dothidea* compared with the bacterial cells (≥10^6^ CFU mL^–1^). Similarly, Aiello et al. (2019) found that *P. synxantha* cells exhibited the best effects to inhibit *M. fructigena* and *M. fructicola* compared to culture filtrates. These data clearly indicated that *Pseudomonas* spp. functioned by other mechanisms involving the presence of living cells, but not antibiosis. For example, competing for nutrients and space with pathogens is the principal mode of *P. syringae*, an availably commercial agent Bio-Save (Bull et al., 1998). This mechanism is also true for other antagonistic bacteria *P. fluorescens* and *B. amyloliquefaciens* B4 (Wallace et al., 2017; Ye et al., 2021).

The pathogen’s infection could cause oxidative damage, which has a negative impact on the cytomembrane and can be illustrated by the changes in electrolyte leakage and MDA contents (Zhang et al., 2012). Previous studies demonstrated that the levels of electrolyte leakage or MDA enhanced in plant tissues infected with pathogens, such as in peach fruit infected with *M. fructicola*, and in apple leaves inoculated with *Glomerella cingulata* (Zhang Q. M. et al., 2016; Ji et al., 2020). Our present research also showed that the levels of electrolyte leakage and MDA increased greatly in apple fruit with only *B. dothidea* infection from 24 to 120 hpi, whereas *P. syringae* B-1 treatment reduced the electrolyte leakage and MDA contents in fruit after *B. dothidea* inoculation. Therefore, these results indicated antagonists were effective in delaying MDA accumulation and mitigating electrolyte leakage in fruit (Zhang Q. M. et al., 2016; Ji et al., 2020).

In addition, oxidative damage also can be induced by the excessive production of ROS in cells (Glazebrook, 2005). It has been reported that ROS is a harmful substance produced during pathogens infection and that the levels of ROS correlate with the severity of disease symptoms (Mittler et al., 2004). In this research, our data indicated that strain B-1 treatment effectively reduced H_2_O_2_ aggregation in response to *B. dothidea* infection. Thus, we speculated that strain B-1 played the antioxidant function, hence alleviating subsequence oxidative damage to apple fruit and conferring resistance to *B. dothidea*. This result agrees with the report of Ji et al. (2020) where the authors reported that *B. licheniformis* W10 treatment reduced disease severity and ROS aggregation in nectarine fruit caused by *M. fructicola*.

Various antioxidant enzymes have vital functions in balancing ROS levels and inducing plant resistance responses (Mittler et al., 2004). Numerous studies have demonstrated that the activation of POD, CAT, SOD, and APX participated in the reaction of plant defense (Zhang Q. M. et al., 2016; Ji et al., 2020; Huang et al., 2021a). For example, in loquat fruit, *B. amyloliquefaciens* B4 increased the defense reaction against various pathogens, and the activity of POD was greatly higher than the control (Ye et al., 2021). In apple fruit, *M. guilliermondii* Y-1 improved three antioxidant enzyme activities and the total antioxidant capacity, thereby correspondingly increasing the fruit resistance to apple ring rot (Huang et al., 2021b). This finding revealed that strain B-1 treatment could trigger the activation of the fruit defense response and mitigate its oxidative damage against *B. dothidea* infection.

In addition to the antioxidant enzymes, the defense-related enzymes, secondary metabolites, and host-resistance proteins are all involved in plant disease resistance (Huang et al., 2021b; Zhang et al., 2021). PAL participates in the biosynthesis of phenolics and lignin in plant tissues, and PPO produces quinines that can inhibit or kill invading pathogens (Mayer, 2006; Romanazzi et al., 2016). Our results also indicated that *P. syringae* B-1 treatment activated those two enzymes, which is consistent with Zhang Q. M. et al. (2016) who reported that PAL and PPO were potentially induced by the antagonist *S. rochei* A-1 in apple fruit. Similarly, those two enzyme activities were also promoted by *Pichia membranifaciens*, which was related to enhancing peach resistance to Rhizopus rot (Zhang et al., 2020). In addition, CHI and GLU are other key enzymes involved in plant defense response by disrupting the cell wall structure of pathogens (Romanazzi et al., 2016). Liu et al. (2021) stated that the activities and gene expression of both GLU and CHI were upregulated to decrease the lesion diameter of Alternaria rot in pear fruit. In this study, we also indicated here that GLU and CHI were markers of plant defense response, due to their activities and gene expression levels being enhanced and could persist in strain B-1 treated fruit. These data in our research demonstrate that the induced resistance against *B. dothidea* may be the main action mechanism of strain B-1.

Phenolics and lignin are the pivotal metabolic products in the phenylpropanoid metabolic pathway, which can strengthen the structure of wound tissues and effectively prevent the invasion of pathogens (Jiang et al., 2019). Our results indicated that *P. syringae* B-1treatment induced the increase of total phenolics and lignin contents in apple fruit throughout the storage. Our results are also consistent with Li et al. (2019), where they found that β-Aminobutyric acid could stimulate total phenolics and lignin contents against *Gilbertella persicaria* infection in red pitaya fruit. Moreover, MeJA could promote wound healing by activating total phenolics and lignin contents in harvested kiwifruit (Wei et al., 2021).

In the plant defense system, SA is a critical regulatory signal molecule, and recent research indicated that it participated in the resistance response against *B. dothidea* in apple fruit (Zhao et al., 2020). In our study, MeJA contents changed slightly after *P. syringae* B-1 treatment in apple fruit; however, SA contents were induced by stain B-1. These results suggested that the SA pathway may play an important role in apple fruit resistance induced by strain B-1. Based on our data, we also found strain B-1 treatment upregulated the transcript levels of *MdPR1* and *MdPR5*, which are the key molecular markers correlating with SA signaling activation (Zhang Q. M. et al., 2016; Huang et al., 2021a). Furthermore, strain B-1 also activated *MdSID2* and *MdPAD4* expression in apple fruit. *MdSID2* performs a vital role in the aggregation of SA signal, and *MdPAD4* contributes to regulating plant defense against pathogen infection (Wiermer et al., 2005; Zhao et al., 2020). These findings further exhibited that SA is involved in apple fruit resistance induced by strain B-1, and similar results were also reported by Huang et al. (2021a). Their research showed that butylated hydroxytoluene effectively controls apple ring rot mainly by activating the SA signaling pathway.

## 5. Conclusion

In our research, a newly isolated *P. syringae* strain B-1 exhibited a strong efficiency against postharvest apple ring rot. Application of strain B-1 suspension at 1 × 10^8^ CFU mL^–1^ reduced the incidence and lesion diameter of apple ring rot *in vivo*. The investigation of the mode of action demonstrated that strain B-1 not only shows the antifungal ability and activates the defense enzymes but also promotes the accumulation of disease-resistant substances and upregulates the transcript levels of PR genes by activating the SA signaling pathway. These results indicate that *P. syringae* strain B-1 has an enormous potential in controlling *B. dothidea* rot during the storage of apple fruit. Further exploration of the application technologies of strain B-1 is under investigation by our team, such as evaluating the tolerance of strain B-1 to fungicides used on apple fruit and the control efficiency of their combined treatments against *B. dothidea*.

## Data availability statement

The original contributions presented in this study are included in the article/supplementary material, further inquiries can be directed to the corresponding author.

## Author contributions

ZS: conceptualization, data curation, and writing—original draft. BH: methodology, investigation, and writing—original draft. CuW: formal analysis and data curation. SL: methodology and data curation. YX: methodology and investigation. BL: funding acquisition and writing—review and editing. CaW: supervision, funding acquisition, and writing—review and editing. All authors contributed to the article and approved the submitted version.
